# The Dark Side of Multimedia Devices: Negative Consequences for Socioemotional Development in Early Childhood

**DOI:** 10.3390/children10111807

**Published:** 2023-11-14

**Authors:** Bruno Rocha, Laura I. Ferreira, Cátia Martins, Rita Santos, Cristina Nunes

**Affiliations:** 1 Department of Psychology and Educational Sciences, Campus de Gambelas, Universidade do Algarve, 8005-135 Faro, Portugal; bmrocha@ualg.pt; 2 Psychology Research Centre (CIP), Universidade do Algarve, 8005-135 Faro, Portugal; liferreira@ualg.pt (L.I.F.); csmartins@ualg.pt (C.M.); rasantos@ualg.pt (R.S.)

**Keywords:** behavioural problems, child, emotional adjustment, infant, preschool, multimedia devices, screen time, socioemotional development

## Abstract

There is growing concern about the relationship between screen use by young children and negative effects on their development, as children with developmental and socioemotional impairments tend to have difficulties in their relationships and increased academic problems. The aim of our study was to analyse the relationship between the use of multimedia devices and paediatric symptoms in children below 5 years old. Data from 534 Portuguese parents of children aged from 18 to 57 months were collected via a self-report questionnaire. Children’s daily exposure to multimedia devices was nearly 2 h per day. Paediatric symptoms were positively associated with watching television and screen time and negatively associated with parents’ working hours. Touchscreen use was more frequent among girls and older children. Parents’ working hours comprised the most significant predictor of paediatric symptoms. Our findings reinforce past findings on the adverse links between the use of multimedia devices and paediatric symptoms and also highlight the influence of other variables like the child’s age and gender, as well as parental factors. The need to both create a more comprehensive framework regarding the long-term effects of multimedia device use and delineate effective strategies for prevention and intervention with parents and children is discussed.

## 1. Background

The term “multimedia devices” refers to all tools, such as televisions (TVs), mobile phones, tablets, computers, and any other electronic equipment, that allow interaction with a screen. These devices have rapidly become part of everyone’s daily life, including all the young children who use them in their routine from an early age [1,2]. The growing use of multimedia by children has been receiving particular attention regarding its impacts on child development [3].

Although the use of these devices can be claimed as a way for parents to calm their child [4], and these devices can have benefits in terms of learning when consumed in moderate “dosages” [5,6], these devices’ excessive use represents a risk factor at many levels. For example, a high use of multimedia devices has been associated with a higher propensity for future metabolic and cardiovascular diseases, childhood obesity, and low physical activity [7,8]. In addition to physical health, the current concerns about the increasing use of electronic devices among young children are linked to their impact on the psychological and socioemotional development of these children.

The existing literature in this field has identified several negative effects of multimedia device use on child development, such as worse sleep quality [9], fewer academic skills [10], delayed language and cognitive development [11,12,13,14], externalising problems [15], lower levels of happiness [16], and a higher occurrence of mental health disorders such as anxiety and depression [17].

Recently, Madigan and colleagues [18] conducted a cohort study that longitudinally assessed 2441 children at 24, 36, and 60 months in terms of their screen time and development and found that children with more screen time at 24 and 36 months reported worse markers of global development at 60 months. Specific to socioemotional development, a longitudinal study of 960 4-year-old Norwegian children, followed up at ages 6 and 8, which aimed to examine the bidirectional relationships between screen use and emotional understanding, concluded that more screen time at the age of 4 was associated with lower emotional understanding at the age of 6 [19]. This was in line with the existing scientific knowledge at the time. Moreover, the use of multimedia devices has also been pointed out as a source of harm to parent–child interactions due to children’s persistent and challenging requests to access these devices, with this behaviour appearing similar to addictive behaviour [20].

Another investigation [21], which aimed to assess the associations between exposure to multimedia devices and behavioural and emotional problems and language development in a sample of 161 children aged between 18 and 36 months, revealed that children exposed to these devices are more likely to present with emotional problems, such as anxiety, depression, aggressive behaviour, somatic symptoms, and social withdrawal [21]. Later, in 2021, Niiranen et al. [22] sought to longitudinally examine the effect of the frequency of electronic media use at 18 months on the psychosocial wellbeing of 699 children at the age of 5. The findings of this study provide evidence that a greater use of multimedia devices can be associated with attentional deficits, concentration difficulties, an increased risk of hyperactivity, and socioemotional problems, including impulsivity, conduct problems, and impairments in interpersonal relationships with peers [22].

Some authors propose that the negative effects of screen exposure may be due to children spending less time on social activities that allow them to develop their social and communication skills. Thus, increased exposure to multimedia and reduced in-person socialisation may result in less collaboration and interaction skills, impairing a child’s emotional, social, and behavioural development [10,21].

Considering the existing data, an important factor is related to the “dosage” of time spent on multimedia devices. On this topic, the World Health Organization (WHO), the American Academy of Paediatrics (AAP), and the SBP (Brazilian Society of Paediatrics) have established guidelines recommending the non-use of multimedia devices by children up to 2 years of age, and that from 2 to 5 years of age, they should not be exposed to more than 1 h of screen time per day [13,23,24]. As an exception, children aged between 18 months and 2 years may be exposed to screens when participating in video calls with their relatives and/or when using electronic devices with parental supervision [23], with the content being educative and compatible with their age [25].

In contrast to these guidelines, empirical studies on screen time have shown values above the suggested values [13,24,26,27,28]. Early childhood sample studies have found that some children exceed 1 h of screen time starting at 18 months old, and that this time increases over subsequent years [2,20]. This growing and alarming use has been noted in studies around the world.

In a study, Dutch children under 7 years old spent 51 min of their day watching TV and 11 min on other electronic devices [29]. In the United Kingdom, it has been seen that 75.2% of children aged from 6 to 36 months used multimedia devices for, on average, about 24 min per day [30], and that half of the 2 year olds had occasional access to the Internet [10]. Also, in India, the average screen time was 2.39 h per day in children under 5 years old [14]. In Portugal, two studies recently sought to assess screen usage time among children. One of them found that children aged between 3 and 5 years spent an average of 154 min per day in front of screens [31], and the other study, with a sample of children under 5 years old, reported an average of 85 min of TV and 57 min of touchscreen use per day [32]. The multimedia devices most frequently used by children consist of TV, followed by touchscreen devices such as smartphones or tablets [11,27,33].

Despite this evidence, precise knowledge around the harmful effects of the use of multimedia devices on socioemotional early year development is still scarce, particularly in Portugal. Thus, more research is needed into the negative consequences of this use, since it has been rising over the last few years. Therefore, the following aims were addressed: (1) to explore the relationship between the use of TV and touchscreen devices, and the presence of psychosocial symptoms in children aged between 18 and 60 months; (2) to analyse the relationship between the use of multimedia devices, psychosocial symptoms in children, and sociodemographic variables (parents and children); and (3) to analyse the predictive contribution of sociodemographic variables and screen time to children’s psychosocial symptoms.

## 2. Materials and Methods

The present study was a quantitative, cross-sectional, and descriptive empirical study, with a simple retrospective investigation plan [34]. 

### 2.1. Participants

A total of 340 parents of children aged from 18 to 57 months (M = 25.33, SD = 10.44), mainly boys (54.1%), from different regions of Portugal, agreed to participate in this study (Table 1). Most parent participants were mothers (86.5%), married (82.6%), and ranging from 22 to 50 years old (M = 36.36, SD = 4.58), with one or two children (M = 1.59, SD = 0.65). Concerning parents’ educational level, 65.9% completed a university degree, while 34.1% reported a lower school degree. Most of the parents were employed (87.9%) and worked a mean of 38.48 h per week (SD = 8.62, range = 0–63).

### 2.2. Measures

Information about the use of electronic devices was obtained via a self-report questionnaire adapted from Nikken and Schols [29] about the use of electronic media devices. Items included questions about sociodemographic characteristics of the parents (i.e., gender, age, marital status, education, and employment status), the children’s sociodemographic characteristics (i.e., gender, age), and questions related to the frequency of electronic device use by the child (devices available and screen time).

Data about social and emotional problems in children were collected using the Portuguese version of the Preschool Paediatric Symptom Checklist (PPSC) [35,36]. The PPSC is a brief social/emotional screening instrument for children aged from 18 to 60 months, with a total of 18 items. The internal consistency reliability of the PPSC was *α* = 0.80. Higher scores indicate higher levels of social/emotional problems.

### 2.3. Data Collection

Data collection took place online via Google Forms during May 2020 with Portuguese parents. The study was approved by the Ethics Committee of ARS Algarve I.P. (Proc Nº. 4/2020). Parental informed consent was obtained from all the participants who had volunteered for the study, with no compensation offered. In cases where parents had more than one child, the questions always pertained to their youngest.

### 2.4. Data Analysis

Data analysis was performed with SPSS software (v.28 IBM SPSS Inc., Chicago, IL, USA). Statistical assumptions for parametric analyses were checked according to Tabachnick and Fidell’s [37] recommendations. For descriptive statistics, the mean, standard deviation, and range values are presented. To assess internal consistency, Cronbach’s alpha was computed, with values above 0.70 considered acceptable and illustrating a good level of internal consistency [38].

A *t*-test for independent groups was conducted to analyse the differences between groups (i.e., parents’ academic level). The homogeneity of variances was calculated using Levene’s test and assumed if the *p*-value was greater than 0.05. When the assumption of homogeneity was not met, the correct version (i.e., Welsch) was used [39]. Cohen’s *d* was calculated as a measure of effect size, and values of 0.20 were considered as low, 0.50 as moderate, and 0.80 as high.

Hierarchical regression analyses with two steps (the “Enter” method) were conducted to determine the predictors of socioemotional problems in children (i.e., the child’s gender, parents’ working hours, and total screen time). *R*^2^ and Δ*R*^2^ were analysed according to *p*-values (*p* ≤ 0.05), and the contribution level was represented by the *β* value.

## 3. Results

### Use of Multimedia Devices

All parents indicated they had a TV (100%) and almost all had a smartphone or tablet (97.1%) at home. Table 2 displays the use of multimedia devices among children.

Most of the participants had let their children watch TV (91.80%) a mean of 83.94 min per day (SD = 79.11, range = 0–600). Regarding smartphone use, 79.70% indicated that the child used it, mainly with adult supervision (67.90%). Globally, the participants also indicated that the child used a touchscreen device (77.60%) a mean of 42.48 min per day (SD = 52.52, range = 0–360). 

Regarding paediatric symptoms, the results varied between 0 (zero) and 24 symptoms, with a mean of 5.98 (SD = 3.97, range = 0–24), with boys reporting slightly higher levels (M = 6.02, SD = 4.11) than girls (M = 5.94, SD = 3.80); however, the difference was not statistically significant (*t* [338] = 0.20, *p* = 0.843, *d* = 0.02).

As Table 3 shows, there were small, significant positive correlations between the paediatric symptoms and TV time (*r* = 0.18, *p* ≤ 0.01) and touchscreen time (*r* = 0.12, *p* ≤ 0.05), and a negative correlation with parents’ working hours (*r* = −0.16, *p* ≤ 0.01).

The results show that children who watched TV also used touchscreen devices (*r* = 0.17, *p* ≤ 0.01) and were older (*r* = 0.23, *p* ≤ 0.01). TV time was positively associated with the use of touchscreens (*r* = 0.13, *p* ≤ 0.05), touchscreen time (*r* = 0.34, *p* ≤ 0.01), and children’s age (*r* = 0.21, *p* ≤ 0.01) and was negatively associated with parents’ academic level (*r* = −0.11, *p* ≤ 0.05). The use of touchscreens was more frequent in girls (*r* = 0.14, *p* ≤ 0.01) and in older children (*r* = 0.34, *p* ≤ 0.01). Touchscreen time was correlated negatively with parents’ academic level (*r* = −0.19, *p* ≤ 0.01).

Regarding the variable of the child’s gender (Table 4), comparisons were performed, and boys were reported as watching more TV (*X^2^* = 0.11, *p* = 0.732, *V* = 0.02), although this different was neither significative nor had a relevant effect size, and girls were reported as using touchscreens more (Girls: Yes = 84.0%, No = 16.0%; Boys: Yes = 72.3%, No = 27.7%), statically significative with a small effect size (*X^2^* = 6.65, *p* = 0.0.10, *V* = 0.14). Girl’s scores for touchscreen time (*t* [338] = 0.134, *p* = 0.134, *d* = −0.16) were higher than boys’, and boys showed a higher amount of TV time (*t* [338] = 1.02, *p* = 0.307, *d* = 0.11), but neither difference is significative nor has a relevant effect size.

Regarding parents’ academic level (i.e., those with and without a university degree; Table 5), the results revealed that children watched TV more than they used touchscreens (e.g., mobile phones, tablets). There were low-to-moderate differences in total screen time (*t* [163.60] = 3.18, *p* = 0.002, *d* = 0.42), in TV time (*t* [188.67] = 3.18, *p* = 0.056, *d* = 0.24), and touchscreen time (*t* [166.31] = 3.07, *p* = 0.003, *d* = 0.40). Children with parents with no university degree show higher levels of use (total screen time: M = 141.16, SD = 144.80; TV time: M = 96.30, SD = 91.60; touchscreen time: M = 56.11, SD = 65.80) than children whose parent had a university degree (total screen time: M = 94.22, SD = 91.26; TV time: *M* = 77.55, *SD* = 71.19; touchscreen time: M = 35.42, SD = 42.60).

Considering the correlations found, we analysed the predictors of paediatric symptoms in two steps (Step 1 = control variables and Step 2 = main effects; Table 6). The results showed that both models were significant, although in Step 2, the change was not significant (Step 1: *R*^2^ = 0.03, *p* ≤ 0.05; Step 2 = 0.04, *p* ≤ 0.05; Δ*R*^2^ = 0.01, *p* = 0.170). In both situations, the predictor with the most significant contribution was the parents’ working hours (*β* = −0.16, *p* ≤ 0.05). Also, the total screen time made a near-significant contribution (*β* = −0.10, *p* ≤ 0.10).

## 4. Discussion

The present study aimed to describe the use of multimedia devices in children aged from 18 to 57 months, analyse its relationship with sociodemographic characteristics and paediatric symptoms, and examine the predictors of multimedia device usage. These objectives were driven by the need to identify the harmful aspects of screen exposure in children’s development, a growing concern today [3,17].

The data obtained indicate that selected Portuguese children were exposed to TV for approximately 84 min per day and approximately 43 min to touchscreens, resulting in a total average exposure of nearly 2 h per day to multimedia devices. The numbers reported in our sample exceed the recommendations that children under the age of 2 should not be exposed to screens, and that children aged from 2 to 5 should have no more than 1 h of total screen time per day [13,23,24]. The results from the present sample are higher than the screen time durations found in studies with children in the United Kingdom [30] and Dutch children [29], but they are similar to the findings of most previous investigations [2,22]. This pattern coincides with the situation of children in India [14] and Portugal, as demonstrated by the data from Rocha and Nunes [32], which indicated exposure to 85 min of TV and 57 min of touchscreen use per day. Similar to the existing scientific evidence, this study’s results reflected that TVs and touchscreens were identified as the most commonly used multimedia devices [11,27,33], demonstrating consistency in the types of devices to which children are exposed, regardless of cultural background [2,22].

These prominent levels of multimedia device usage at an early age are alarming, especially considering both that the trend increases with age [22] and that screen time is consistently associated with significant negative risks for child development. A recent study by Takahashi et al. [40] with a sample of 7097 children aged from 1 to 4 years revealed that increased screen time at 1 year of age was associated with a higher risk of developmental problems in communication, problem solving, and social skills at 2 years of age, and with communication and problem-solving issues at 4 years old. The authors underscored the importance of managing the use of these devices while also learning to achieve positive benefits from them. In other words, in a world where limiting screen access is increasingly challenging, it is essential to ensure that screen time includes educational content that benefits a child’s development [40].

Regarding our examination of the associations between multimedia device use, psychosocial symptoms in children, and sociodemographic factors, it is noteworthy that there was a positive correlation, albeit small, between paediatric symptoms and TV and touchscreen exposure, and a nearly significant contribution of screen time to paediatric symptoms. These findings reinforce what was already described in most studies on this topic regarding the harmful effects of excessive exposure to these devices [20,21,41,42,43]. Furthermore, the use of multimedia devices also appeared to be more associated with girls and older children, findings that cohere with past empirical research [22,44]. This information can be valuable for preventive actions and future interventions.

Also, a negative correlation was found between paediatric symptoms and parents’ working hours, indicating that more symptoms were associated with children whose parent worked fewer hours. Moreover, parents’ working hours appeared as the predictor with the most significant contribution to multimedia device usage. These data are new, interesting, and should be further explored. They also underscore the need to include parental practices as a mediating or moderating factor that can influence the relationship between screen use and developmental problems. These parents, despite being with their children, may face difficulties in exercising or finishing household tasks, leading them to give their children these devices to establishing a kind of parent–child interaction and occupy the child’s leisure time at home [22,45]. Speculatively, this could result in more screen time for the whole family and contribute to more paediatric symptoms. Future investigators might also measure family dynamics to attempt to clarify this point.

Concerning parental factors, the use of touchscreens has been related to children with parents with lower academic levels [31,46]. Still, our results indicate a higher mean of multimedia device usage in children of parents without an academic degree. Increased electronic device usage among these children could be driven by the fact that families with lower academic backgrounds often have fewer resources to provide traditional educational materials and/or could be due to the parents relying on them for various tasks, such as communication, information seeking, and entertainment [4,20]. This suggests that these parents are more susceptible to increasing the risks to their children’s development through these means, stressing the importance of family support policies in this matter [32,47].

In general, the screen time among the children in our sample far exceeded the recommendations set as a guideline for healthy child development. This excessive screen time was associated with more paediatric symptoms, which may potentially lead to more severe physical and psychological conditions in the future [41]. These conditions can include obesity and cardiovascular diseases [8,36], learning difficulties, and cognitive development delays [10,14], as well as psychological disorders such as anxiety and depression [17,42,43,48]. One mechanism that may underlie this relationship between paediatric symptoms and the use of multimedia devices is that the amount of time that children dedicate to these media reduces the time that they spend on other educational and developmental activities, such as playing, interaction with family members or peers, reading, or drawing [22].

For this reason, and accounting for the fact that stimulation in the child’s environment is a determining factor in healthy growth [21], there is a need for a greater focus on parental education, such that parents are both aware of scientific health organisation recommendations and have some support in planning additional healthy activities for their children when the parents are at home that do not involve exposure to screens [45,49], especially for parents with lower academic levels. This can include promoting outdoor activities, identifying alternative and constructive activities, and establishing a well-defined schedule to limit children’s use of touchscreens and their watching TV.

Taken together, our findings emphasise the need for parenting and child development support programs focused on promoting well-adjusted educational engagement with multimedia devices [49], thus preventing later negative outcomes.

Nevertheless, despite this study’s positive contributions, there are some noteworthy limitations. First, the sample was highly homogeneous, and it would have been beneficial to include more fathers, parents with more children, and parents with a more diverse range of socioeconomic and educational levels. Second, the sample was collected online through voluntary responses to a questionnaire that was shared on social networks and posted on websites for parents, which, ironically, ended up restricting the responses to a group of people who use these platforms. The exclusive use of self-report questionnaires was another limitation to recognise, as it probably triggered response bias. Finally, inclusion of additional measures related to family dynamics and parenting practices was lacking. Future researchers might recruit a more heterogeneous sample and employ longitudinal designs to allow for a more in-depth examination of the psychological consequences of multimedia device usage over time. Also, future studies might explore a possible bidirectional link between paediatric symptoms and screen time via other methodologies, such as qualitative interviews with parents or observational measurements.

In summary, this study has helped to both shed light on the effects of multimedia device usage on paediatric symptoms in children aged from 18 to 57 months and show how these effects are influenced by variables related to a child’s age and gender, as well as parental factors such as working hours. The research in this area should continue in order to provide more nuanced insights into how multimedia device usage impacts children’s psychosocial development, and how related psychosocial problems can be prevented.

## 5. Conclusions

This study highlights the importance of addressing the potential negative consequences of excessive screen time in young children and the role of sociodemographic factors and parental practices in influencing screen usage. The children in the sample were exposed to approximately 2 h of screen time per day, exceeding the recommended limits. Notably, screen time was higher among girls, older children, and those whose parents had lower academic levels.

Our findings revealed a clear association between excessive screen time and adverse health and developmental problems. This underscores the importance of parental education and support in encouraging more balanced screen usage and providing alternative activities for children, to promote their wellbeing.

## Figures and Tables

**Table 1:** Participants’ sociodemographic characteristics.

	Parents (*n* = 340)				Children		
	%	M	SD	Range (Years)	M	SD	Range (Months)
Age		36.36	4.58	22–50	25.33	10.44	18–57
Gender							
Female	86.5						
Male	13.5						
Marital status							
Married	82.6						
Not married	17.4						
Educational level							
Lower school degree	34.1						
University degree	65.9						
Employment status							
Unemployed	12.1						
Employed	87.9						
Number of children per family					1.59	0.65	1–2
Working hours per week		38.48	8.62	0–63			

Note. M = mean, SD = standard deviation.

**Table 2:** The use of television and touchscreen devices (smartphone or tablet) in children from 18 to 60 months (*n* = 340).

Children’s Use of Multimedia			%	M	SD	Range
Television	Watch TV	Yes	91.80			
		No	8.20			
	Time (daily use min/day)			83.94	79.11	0–600
Smartphone	Use a device	Yes	79.70			
		No	20.30			
	With adult supervision	Always or most of the time	67.90			
		Often	12.20			
		Sometimes	12.90			
Touchscreen	Use a device	Yes	77.60			
		No	22.40			
	Time (daily use min/day)			42.48	52.52	0–360
Total screen exposure				110.24	114.40	0–920

Note. M = mean, SD = standard deviation.

**Table 3:** Correlations between the use of television and touchscreen devices (smartphones or tablets), paediatric symptoms, and sociodemographic features (*n* = 340).

	1	2	3	4	5	6	7	8
1. Paediatric symptoms	1							
2. Watch TV	−0.01	1						
3. TV time	0.18 **	0.32 **	1					
4. Use touchscreen	0.07	0.17 **	0.13 *	1				
5. Touchscreen time	0.12 *	0.12 *	0.34 **	0.44 **	1			
6. Child’s age	0.04	0.23 **	0.21 **	0.34 **	0.26 **	1		
7. Parents’ academic level	−0.08	−0.10	−0.11 *	−0.07	−0.19 **	−0.07	1	
8. Parents’ working hours	−0.16 **	−0.07	−0.05	−0.05	0.02	−0.10	0.09	1

Notes: for the variables “Watch TV” and “Use touchscreen”, the codification is No = 1, Yes = 2; * *p* ≤ 0.05. For “Child’s gender”, Boy = 1, Girl = 2; ** *p* ≤ 0.01.

**Table 4:** Comparisons of TV use and time, and touchscreen use and time regarding the variable of the child’s gender (*n* = 340).

Domains		Boys (*n* = 184)		Girls (*n* = 156)		*t* (*df*)	*p*	*d*
		M	SD	M	SD			
TV time		87.99	85.40	79.18	70.97	1.02 (338)	0.307	0.11
Touchscreen time		38.55	53.05	47.12	51.68	−1.50 (338)	0.134	−0.16
		*f*	%	*f*	%	*X* ^2^	*p*	*V*
Watch TV	No	16	8.7	12	7.7	0.11	0.737	0.02
	Yes	168	91.3	144	92.3			
Use touchscreen	No	51	27.7	25	16.0	6.65	0.010	0.14
	Yes	133	72.3	131	84.0			

Notes. M = mean; SD = standard deviation; *t* = *t*-test; *p* = *p*-value; *d* = Cohen’s *d* effect size; *f* = count; *X*^2^ = Chi square; V = Cramer’s V effect size.

**Table 5:** Mean comparisons of screen time, TV time, and touchscreen time regarding parents’ academic level (*n* = 340).

	No University Degree (*n* = 116)		University Degree (*n* = 224)		*t* (*df*)	*p*	*d*
	M	SD	M	SD			
Total screen time	141.16	144.80	94.22	91.26	3.18 (163.60)	0.002	0.42
TV time	96.30	91.60	77.55	71.19	1.92 (188.67)	0.056	0.24
Touchscreen time	56.11	65.80	35.42	42.60	3.07 (166.31)	0.003	0.40

Notes. M = mean; SD = standard deviation; *t* = statistic score; *p* = *p*-value; *d* = effect size.

**Table 6:** Summary of hierarchical regression analyses predicting paediatric symptoms in the total sample (*n* = 340).

		Paediatric Symptoms			
	*R^2^*	∆*R^2^*	Change in *F*	*β*	*t*
Step 1—Control variables	0.03 *	-	4.09 *		
Child’s gender				−0.01	0.02
Parents’ working hours				−0.16 **	−2.86 **
Step 2—Main effects	0.04 *	0.01	3.17		
Child’s gender				−0.00	−0.07
Parents’ working hours				−0.16	−2.81 **
Total screen time				0.10	1.78 ^#^

^#^ *p* < 0.10; * *p* ≤ 0.05; ** *p* ≤ 0.01.

## Data Availability

The data can be made available for consultation from the corresponding author upon request.
